# EEG Microstates Temporal Dynamics Differentiate Individuals with Mood and Anxiety Disorders From Healthy Subjects

**DOI:** 10.3389/fnhum.2019.00056

**Published:** 2019-02-26

**Authors:** Obada Al Zoubi, Ahmad Mayeli, Aki Tsuchiyagaito, Masaya Misaki, Vadim Zotev, Hazem Refai, Martin Paulus, Jerzy Bodurka, the Tulsa 1000 Investigators, Robin L. Aupperle

**Affiliations:** ^1^Laureate Institute for Brain Research, Tulsa, OK, United States; ^2^Department of Electrical and Computer Engineering, University of Oklahoma, Tulsa, OK, United States; ^3^Japan Society for the Promotion Science, Tokyo, Japan; ^4^Research Center for Child Development, Chiba University, Chiba, Japan; ^5^Stephenson School of Biomedical Engineering, University of Oklahoma, Norman, OK, United States; ^6^Oxley College of Health Sciences, University of Tulsa, Tulsa, OK, United States

**Keywords:** EEG microstate, brain, mood and anxiety disorders, temporal dynamic, transition probabilites

## Abstract

Electroencephalography (EEG) measures the brain’s electrophysiological spatio-temporal activities with high temporal resolution. Multichannel and broadband analysis of EEG signals is referred to as EEG microstates (EEG-ms) and can characterize such dynamic neuronal activity. EEG-ms have gained much attention due to the increasing evidence of their association with mental activities and large-scale brain networks identified by functional magnetic resonance imaging (fMRI). Spatially independent EEG-ms are quasi-stationary topographies (e.g., stable, lasting a few dozen milliseconds) typically classified into four canonical classes (microstates A through D). They can be identified by clustering EEG signals around EEG global field power (GFP) maxima points. We examined the EEG-ms properties and the dynamics of cohorts of mood and anxiety (MA) disorders subjects (*n* = 61) and healthy controls (HCs; *n* = 52). In both groups, we found four distinct classes of EEG-ms (A through D), which did not differ among cohorts. This suggests a lack of significant structural cortical abnormalities among cohorts, which would otherwise affect the EEG-ms topographies. However, both cohorts’ brain network dynamics significantly varied, as reflected in EEG-ms properties. Compared to HC, the MA cohort features a lower transition probability between EEG-ms B and D and higher transition probability from A to D and from B to C, with a trend towards significance in the average duration of microstate C. Furthermore, we harnessed a recently introduced theoretical approach to analyze the temporal dependencies in EEG-ms. The results revealed that the transition matrices of MA group exhibit higher symmetrical and stationarity properties as compared to HC ones. In addition, we found an elevation in the temporal dependencies among microstates, especially in microstate B for the MA group. The determined alteration in EEG-ms temporal dependencies among the cohorts suggests that brain abnormalities in mood and anxiety disorders reflect aberrant neural dynamics and a temporal dwelling among ceratin brain states (i.e., mood and anxiety disorders subjects have a less dynamicity in switching between different brain states).

## Introduction

Electroencephalography (EEG) has been used for studying and phenotyping various types of neuropsychiatric and neurodegenerative disorders (Niedermeyer and Da Silva, 2005; Allen and Reznik, 2015; Horvath et al., 2018). Recent efforts aim to discover and provide a cost-effective, reliable markers for aberrant brain activity patterns relevant to major psychiatric disorders. Distinct topographic representation of the EEG, lasting a few dozens of a millisecond, coined an EEG-microstates (EEG-ms), provides an opportunity and a novel tool to discover unique markers of different brain disorders (Khanna et al., 2015). EEG-ms was first introduced by Lehmann et al. (1987), where it was revealed that EEG could be segmented in a few quasi-stable states (microstates). The segmentation of EEG signals is carried out at extrema points of the EEG global field power (GFP), which can maintain a high signal-to-noise ratio and provide a reliable source for identifying the microstates. Two seminal reviews of EEG-ms were presented in Khanna et al., 2015 and Michel and Koenig (2018).

The functional interpretation of EEG-ms could be explained as coordinated and synchronized neuronal current activity of many neurons that happens to be activated together as demonstrated in previous studies (Khanna et al., 2015; Michel and Koenig, 2018). Thus, a change in the topographies of microstates may be attributed to a change in the orientation or distribution of the current dipoles (Vaughan, 1980; Lehmann et al., 1987). The alteration in the properties of EEG-ms presumably reflects a disruption in the underlying brain networking processes and information flow. Furthermore, spatially independent EEG-ms (Lehmann et al., 1987), and especially temporally independent EEG-ms (Yuan et al., 2012, 2016), were revealed to be correlated with resting state networks (RSNs), measured by functional magnetic resonance imaging (fMRI; Britz et al., 2010; Musso et al., 2010; Yuan et al., 2012). Additionally, other studies reported that EEG-ms are associated with particular mental processes (Brandeis and Lehmann, 1989; Brandeis et al., 1995; Koenig and Lehmann, 1996; Pizzagalli et al., 2000; Michel et al., 2001; Britz and Michel, 2010; Britz et al., 2010, 2014; Michel and Koenig, 2018). The source localization of EEG-ms was investigated in Custo et al. (2017), where authors identified seven microstates (A through G) and localized the source of these microstates. Their results suggest a common activation among those microstates in the brain’s main hubs (like the precuneus, anterior and posterior cingulate cortices, insula, superior frontal cortex, and other brain regions). Therefore, the EEG-ms can characterize network alteration or disruption in brain functionality due to disorders and offer potential biomarkers. Evidence of the relation between mental processes and EEG-ms has led to several works to study EEG-ms properties in neuropsychiatric disorders. Early works of spatially independent EEG-ms focused on schizophrenia and showed moderate to substantial differences in EEG-ms properties between subjects with schizophrenia and healthy groups (Strik et al., 1995; Lehmann et al., 2005; Nishida et al., 2013; Andreou et al., 2014; Tomescu et al., 2014). Other works have also revealed an alteration in EEG-ms for other diseases, like dementia produced by Alzheimer’s (Dierks et al., 1997; Strik et al., 1997; Stevens and Kircher, 1998). Some neuropsychiatric illnesses were also shown to affect certain microstates, including depression (Strik et al., 1995), panic disorder (Kikuchi et al., 2011), narcolepsy (Drissi et al., 2016), multiple sclerosis (Gschwind et al., 2016) and Tourette syndrome (Stevens et al., 1996). In this study, we specifically focused on mood and anxiety disorders, because emotion regulation alterations have been consistently described for these disorders (Campbell-Sills et al., 2006; Aldao and Nolen-Hoeksema, 2010). Major depressive and anxiety disorders share highly overlapping symptoms, altered brain networks and brain region activities [e.g., default mode, executive, salience networks, and prefrontal cortex, cingulate cortex, hippocampus, and amygdala (Ressler and Mayberg, 2007)]. Therefore, detecting and characterizing the dynamics of brain neuronal activity through transient spatial/temporal EEG-ms patterns may provide novel information and improve our understanding of the mechanisms of irregularities in cognitive and emotion processing among psychiatric disorders.

The typical spatially independent EEG-ms analysis is conducted by locating the peaks of the GFP and then clustering EEG points around these peaks. For running such an analysis, the desired number of microstates (clusters) needs to be specified before running the clustering algorithm. A majority of EEG-ms studies have used the four canonical microstates to study group difference (Michel and Koenig, 2018); however, other studies have identified additional microstates besides the four canonical ones (Yuan et al., 2012; Custo et al., 2017). Although using a predefined number of clusters is arguable, it is preferable to compare among different groups. We follow the literature by defining the number of desired microstates (*k* = 4) for both groups (Michel and Koenig, 2018). Several characteristics for EEG-ms can be extracted, such as the average duration, the frequency of occurrence, and transition probabilities. Each property can be interpreted based on the underlying neural activities. For instance, the average EEG-ms duration, which represents the temporal stability of each microstate, while the frequency of EEG-ms occurrence may represent the tendency of microstates to be active. The transition probabilities extract the asymptotic behavior of transitions between microstates (i.e., the likelihood of switching between different microstates). To further examine the dynamics in the EEG-ms sequence, we adopted a new set of features introduced in von Wegner et al. (2017). The work provides an information-theoretical analysis to investigate the dynamics of EEG-ms and to assess temporal dependencies between microstates.

The present study aimed to further explore the possible association among the EEG-ms dynamic patterns and mood and anxiety disorders. The objective of this study was to describe abnormalities of the EEG-ms in mood and anxiety disorders, primarily focusing on both general EEG-ms properties and temporal associations within EEG-ms occurrence sequence and temporal dynamics. As the EEG-ms relates to intrinsic brain functional networks that are active at rest, we hypothesized that there should be significant differences in EEG-ms dynamics between the mood and anxiety group and the healthy control (HC) group.

## Materials and Methods

### Participants

Participants were selected from the first 500 subjects of the Tulsa 1000 (T-1000), a naturalistic study assessing and longitudinally following 1,000 individuals, including healthy comparisons and treatment-seeking individuals with mood disorders and/or anxiety, substance use, and eating disorders (Victor et al., 2018). For this work, the datasets are comprised of 52 HC subjects (28 females) and 61 naive (un-medicated) subjects (38 females) with mood and anxiety disorders. The T-1000 study aims to determine how mood and anxiety disorders, substance use, and eating behavior organize across different levels of analysis with a focus on predictors of long-term prognosis, symptom severity, and treatment outcome. The T-1000 study is conducted at the Laureate Institute for Brain Research. The human data used for the analysis were obtained as part of Tulsa 1000 naturalistic study in large, 1000 participants, psychiatric population. All study procedures were carried out in accordance with the principles expressed in the Declaration of Helsinki. After receiving a complete explanation of the study procedures, all participants provided written informed consent as approved by the Western Institutional Review Board (WIRB) protocol #20101611. Participants received financial compensation for their participation. As described in detail in Victor et al. (2018), the participants in this work were screened on the basis of treatment-seeking history and dimensional psychopathology scores: Patient Health Questionnaire-9 (PHQ-9) ≥10 and/or Overall Anxiety Severity and Impairment Scale (OASIS) ≥8. Each participant underwent approximately 24 hours of testing over the course of 1 year, including a standardized diagnostic assessment, self-report questionnaires, behavioral and physiological measurements indexing RDoC domains, and blood/microbiome collection. A structural MRI, resting-state fMRI, task-based fMRI during reward-related processing, fear processing, cognitive control/inhibition, and interoceptive processing were also collected with simultaneous EEG recording. Please refer to Supplementary Table S1 for detailed information about the demographics of the dataset. In this study, we used the EEG data acquired during resting state fMRI and the self-report questionnaire data.

### EEG Data Acquisition

MRI imaging and simultaneous EEG-fMRI was conducted using a General Electric Discovery MR750 whole-body 3 T MRI scanner with a standard 8-channel, receive-only head coil array. A single-shot gradient-recalled EPI sequence with Sensitivity Encoding (SENSE) was employed for the fMRI acquisition. EEG signals were recorded simultaneously with fMRI using a 32-channel MR-compatible EEG system (Brain Products GmbH) with measuring electrodes arranged according to the international 10–20 system. ECG signal was recorded using an electrode on the subject’s back. In order to synchronize the EEG system clock with the 10 MHz MRI scanner clock, a Brain Products’ SyncBox device was utilized. The EEG acquisition of temporal resolution and measurement resolutions was 0.2 ms (i.e., 16-bit 5 kS/s sampling) and 0.1 μV, respectively. A hardware filtering throughout the acquisition in a frequency band between 0.016 and 250 Hz was applied to EEG signals.

We included EEG data collected from 113 subjects during resting EEG-fMRI run lasting 8 min. The participants were instructed to relax and keep their eyes open and fixate their eyes on a cross displayed on the fMRI stimulus projection screen. For this work, we did not use and analyze fMRI data.

### EEG Data Preprocessing

The following preprocessing steps were performed in BrainVision Analyzer 2 software as described in Mayeli et al. (2016). In short, MRI imaging artifacts were reduced using the average artifact subtraction (AAS) method (Allen et al., 2000) and EEG signals were down-sampled to 250 Hz. Next, band-rejection filters (1 Hz bandwidth) were used to remove fMRI slice selection fundamental frequency (19.5 Hz) and its harmonics, mechanical vibration noise (26 Hz), and AC power line noise (60 Hz). Then, we used a bandpass filter from 0.1 to 80 Hz (48 dB/octave). Cardiobalistic artifacts (BCG) also were removed using AAS (Allen et al., 1998). The independent component analysis (ICA, Infomax algorithm; Bell and Sejnowski, 1995) implemented in Analyzer 2 was applied for EEG signals ICA decomposition. The topographic map, power spectrum density, time course signal, energy value, and kurtosis value were used for detecting and removing artifactual ICs, including residual BCG and imaging, ocular and muscle artifacts. Finally, the EEG signal was reconstructed using back-projection (inverse ICA) after selecting ICs related to neural activities.

### EEG Microstates Analysis

The typical spatially independent EEG-ms analysis is conducted by calculating the peaks of the GFP after using average-reference (Michel and Koenig, 2018). Then, EEG time points corresponding to those peaks are fed into modified-clustering algorithms. Two common clustering algorithms have been widely used in the literature: modified-k-means (Pascual-Marqui et al., 1995) and agglomerative hierarchical clustering (AAHC; Murray et al., 2008). Both algorithms compute the template EEG-ms in different ways, but they appear to produce similar results (Murray et al., 2008). In this work, we utilized the AAHC algorithm for segmenting the EEG points after setting the number of desired microstates to *k* = 4. The following steps were required before running AAHC algorithm: first, the GFP for each subject was calculated from a band passed filtered EEG data between 2 and 20 Hz (using FIR with heuristically estimated transition band implemented with pop_eegfiltnew from EEGLAB) as suggested in several EEG-ms studies (Michel and Koenig, 2018). The peaks of GFP were then identified after smoothing GFP with a Gaussian-weighted moving average of 5-time points. Finally, to offer a higher level of accuracy, we randomly selected up to *n* = 10,000 peaks and extracted the corresponding EEG points for later analysis. The selected EEG points were then submitted to the AAHC algorithm to identify the microstates with *k* = 4. Next, the group means of EEG-ms from each group were computed by sorting individual EEG-ms first and then finding the common topographies across all subjects. After that, individual EEG sets were fit-back using the group means topographies. Finally, we extracted the following EEG-ms characteristics from each subject: average lifespan, the frequency of occurrence, and transition probabilities, in addition to the occurrence sequence of each microstate. Also, we conducted a theoretical information analysis described below to examine the temporal dynamics of the EEG-ms.

### Information Theoretical Analysis

Studying the dynamic behavior and the temporal dependencies of EEG-ms sequence may carry useful information that embodies differences in information flow among groups. To do so, we adopted a new set of features introduced by von Wegner et al. (2017). The approach relies on handling the spatially independent EEG-ms as discreet stochastic processes and examines the temporal dependencies in microstates sequence. To elaborate on the set of utilized features, let’s assume a random variable *X*_t_ that represents the state of microstate at time point *t*. The *X*_*t*_ can take one of the possible labels *S*_*i*_ ∈ (*A, B, C, D*), such that *P*(*X*_*t*_ = *S*_*i*_) represents the distribution of the microstates labels across the sequence of EEG-ms. The probability of transition between two states is given as *T*_*ij*_ = *P*(*X*_*t*+1_ = *S*_*j*_|*X*_*t*_ = *S*_*i*_) and the transition matrix is denoted as *T*.

Herein, we assessed the low-order Markovianity of order 0, 1 and 2. That is, EEG-ms were tested to see whether the transition of microstates relied on only the current state [order 0; *P*(*X*_*t*+1_) = *P*(*X*_*t*+1_|*X*_*t*_)], previous state [order 1; *P*(*X*_*t*+1_|*X*_*t*_, *X*_*t*−1_) = *P*(*X*_*t*+1_|*X*_*t*_)] or two previous states [order 2; *P*(*X*_*t*+1_)|*X*_*t*_, *X*_(*t*−1)_, *X*_(*t*−2)_) = *P*(*X*_(*t*+1)_|*X*_*t*_, *X*_(*t*−1)_)]. We also tested whether the transition matrix *T* is stationary by first dividing the data into B overlapping blocks of length L. Then, the transition matrix for each block was assessed against the overall transition matrix. Furthermore, the transition matrix was also tested against the symmetry property i.e., *P*(*X*_(*t*+1)_ = *S*_*j*_ |*X*_*t*_ = *S*_*i*_) = *P*(*X*_(*t*+1)_ = *S*_*i*_|*X*_*t*_ = *S*_*j*_). Finally, the time-lagged mutual information (autoinformation, AIF) was computed for the global sequence of EEG-ms and for individual microstates. AIF examines the amount of information that *X*_(*t*+τ)_ has about *X*_*t*_ with τ is the desired time lag. The higher the value of AIF, the more shared information is carried by *X*_(*t*+τ)_ about *X*_*t*_.

## Results

**Figure 1:**
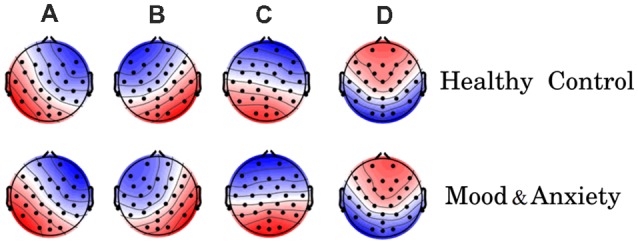
Electroencephalography-microstate (EEG-ms) topographies for both groups [healthy control (HC) group top row, mood and anxiety disorders (MA) group lower row]. The obtained EEG-ms topologies are similar to those reported previously in the literature.

First, we examined the EEG-ms topographies for MA and HC groups. Figure 1 shows the four canonical EEG-ms classes for both groups. We found similar EEG-ms topography templates for both groups (i.e., microstates A through D) and similar to those obtained by previous works (Michel and Koenig, 2018). The performance of the EEG-ms segmentation algorithm is reported in terms of the explained variance, which estimates the portion of EEG point topography that can be explained by the four microstates (Khanna et al., 2014). The explained variance in our case was 82% ± 0.02% for HC and 82% ± 0.01% for MA.

**Figure 2:**
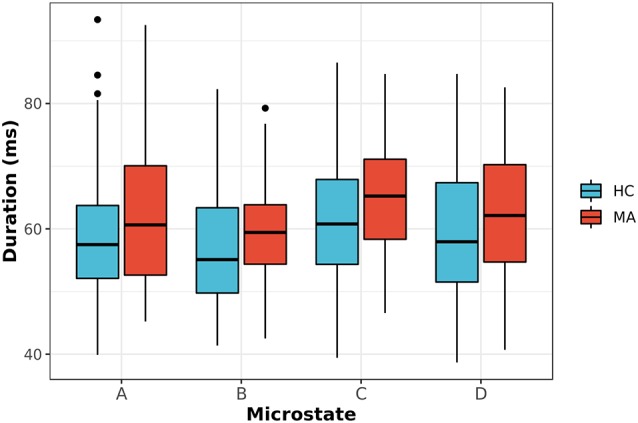
The average duration for EEG-ms classes (A–D) for MA and HC groups (*p*-value corrected for multiple comparisons using the Bonferroni-Holm). The results revealed a trend towards significance for microstate C with *p* = 0.092.

**Figure 3:**
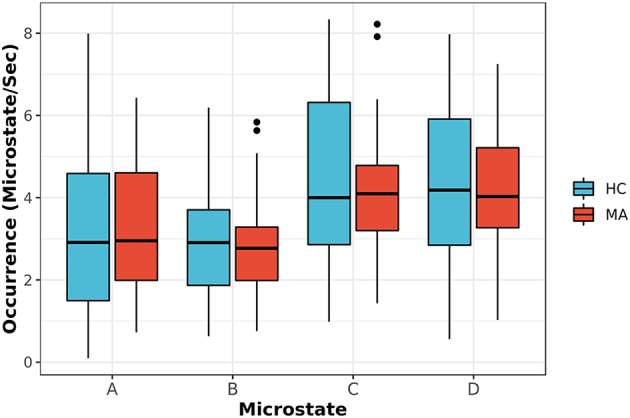
The occurrence frequency of EEG-ms classes (A–D) for both MA and HC groups. For each EEG-ms class, no statistically significant differences among the two groups was found.

Second, the average duration and occurrence frequency were investigated for both groups. Figure 2 shows the average duration for each EEG microstate. The *p*-values for the *t*-test between each microstate were 0.117, 0.042, 0.023 and 0.244 for microstate A, B, C, and D, respectively. After correcting for multiple comparisons using Bonferroni-Holm, the adjusted *p-values* were 0.23, 0.126, 0.092, and 0.244 for A, B, C, and D, respectively. Similarly, the occurrence of each microstate per second was computed for both groups (Figure 3). The results did not reveal any significant difference between the groups.

**Figure 4:**
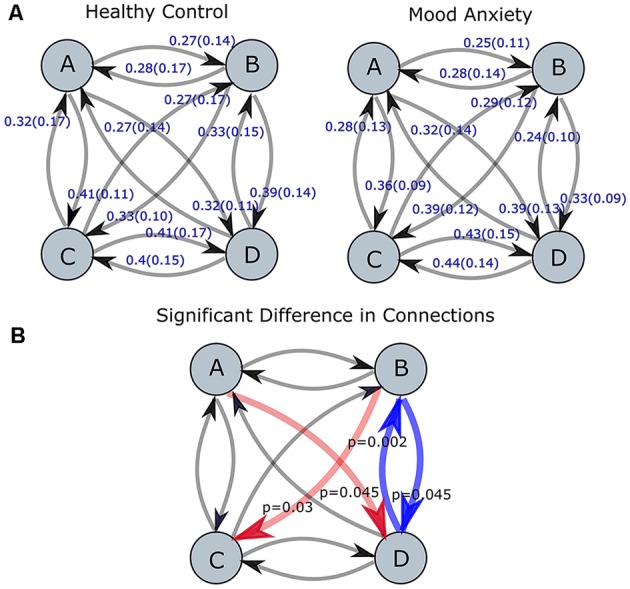
Transition probabilities for MA and HC groups are shown in part **(A)**. The red and blue arrows (red represent an increase, while blue represent a decrease for MA group as compared to HC one) in part **(B)** represent the connections with the statistically significant difference between two groups (*p*-values corrected for multiple comparisons using Bonferroni-Holm). The level of significance was set to *p* < 0.05.

After that, the model of transition among microstates for both groups was investigated and depicted in Figure 4A. The transition probabilities appear to have a normal distribution after checking and using Q-Q and density plots (Ghasemi and Zahediasl, 2012). The statistical analysis of the transition probabilities of groups unraveled a significant difference (Bonferroni-Holm corrected, *p* < 0.05) between HC and MA in four transitions (Tr): from microstate B to D: Tr (B → D), D to B: Tr (D → B), A to D: Tr (A → D) and B to C: Tr (B → C). The statistical analysis for the significant connections was reported in terms of the *t*-test, *p*-value (*p*) and Cohen’s d (*d*) effect size as follows: for Tr (B → D): *t*_(111)_ = 2.69, *p* = 0.045, *d* = 0.51; Tr (D → B): *t*_(111)_ = 3.87, *p* = 0.002, *d* = 0.73; Tr (B → C): *t*_(111)_ = −3.05, *p* = 0.03, *d* = −0.58; Tr (A → D); *t*_(111)_ = −2.88, *p* = 0.045, *d* = −0.54. Figure 4B highlights the transition probabilities that show a statistically significant difference across groups and the direction of change. The associations between these transition probabilities and the symptoms (PHQ-9, RSS, STAI-Trait, STAI-State, PROMIS-Depress, and PROMIS-Anxiety scores) were investigated in Supplementary Table S3 (please refer to the Supplementary Material).

Also, we delved into the EEG-ms temporal dynamic within EEG-ms sequences. For both groups, we assessed the symmetry property of transition matrices and tested for Markovianity of order 0, 1, and 2 properties (Table 1) as described in the information theoretical analysis section. The *t*-test yielded *p-value* which represents the null-hypothesis that subject’s EEG-ms sequence exhibits a low-order Markovian property (e.g., for an order of 0 the transition probability relied on only the current state) or symmetrical transition matrix (e.g., the likelihood of switching from microstate X to Y is not statistically different from the likelihood of switching from Y to X). All tests were conducted at alpha = 0.01 and *p* < 0.05. Table 1 reports the testing results as the ratio of how many subjects showed statistically significant hypothesis (e.g., the EEG-ms sequence exhibits a Markovian property of order 0).

**Table 1:** Markovian property and symmetry assessment for both groups.

	Order 0	Order 1	Order 2	Symmetry
Healthy control	0%	0%	0%	58%
Mood and anxiety	0%	0%	0%	65%

**Figure 5:**
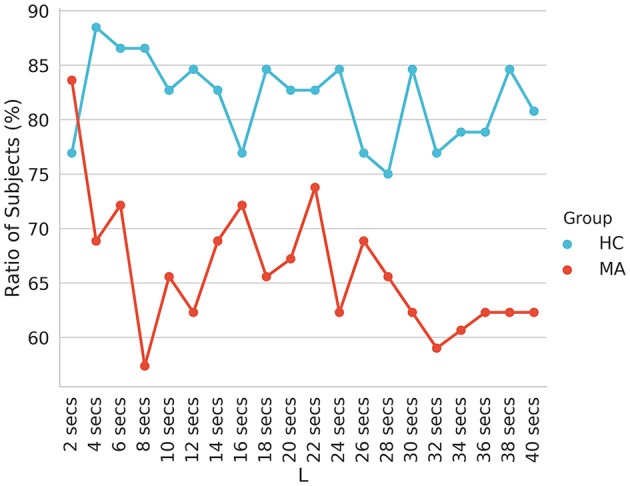
The ratio of subjects with non-stationary transition matrices (*p* < 0.05) of EEG-ms evaluated at different block lengths.

The reported transition probabilities in Figure 4 unveil the overall transition probabilities estimated for the entire recording of EEG (8 min). We further probed the stationary of the transition matrices over a shorter period (i.e., whether the transition matrices remain constant over a short duration). Specifically, we computed the stationary of the transition matrices at period lengths of 2–40 s and reported the ratio of subjects who have statistically significant non-stationary matrices at each period (Figure 5).

**Figure 6:**
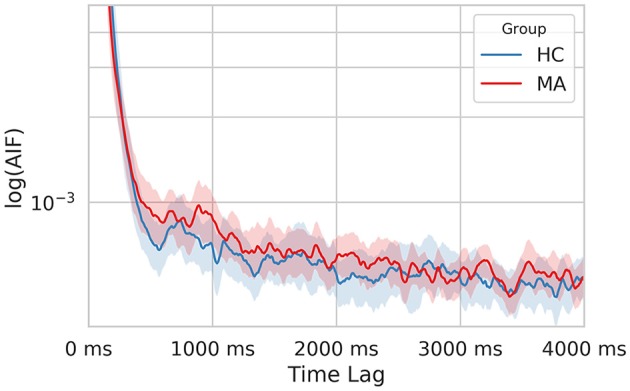
The semi-log time-lagged mutual information plot for the MA and HC groups at different time lags. The shaded area represents the 95% confidence intervals for each group.

**Figure 7:**
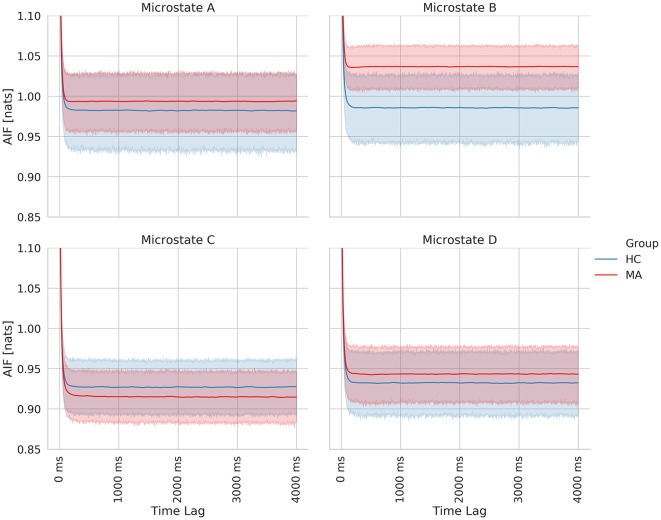
Time-lagged mutual information plots for each class of EEG microstate averaged across subjects of each group. The shaded area represents the 95% confidence for each group.

Finally, we computed the AIF to examine the temporal dependencies in EEG-ms. AIF estimates the amount of information that the appearance of microstates carries given previous information (previous microstates). In other words, it evaluates the memory effect in microstates’ sequence over the shorter duration; the higher the value, the more similar the microstate sequence given the past. By comparing AIF among groups, one can tell whether a certain group has a higher tendency to evoke the same patterns of microstates sequences over and over. Figure 6 shows AIF plot as a function of different time-lags (τ ≤ 4000 ms) for both MA and HC groups. The individual contribution to the overall AIF graph is presented in Figure 7.

## Discussion

In this study, we derived and dissected the EEG-ms from large cohorts of MA and HC individuals. We found the four canonical EEG-ms classes (A through D), which confirms a successful replication of previously reported topographies (Michel and Koenig, 2018). Interestingly, we did not find a specific dissimilarity in the topographies of EEG-ms between HC and MA groups (Figure 1). That is, EEG-ms topographies were stable and robust regardless of the presence of mood and anxiety symptoms. Given that EEG-ms represent spontaneous, synchronized in time, and large spatial-scale cortical neuronal activities (Khanna et al., 2015; Michel and Koenig, 2018), the lack of differences between participant cohorts suggests that there are no major structural cortical changes among groups (Drevets and Raichle, 1992; Mayberg, 2003; Waters and Mayberg, 2017). If EEG-ms topography exhibited significant changes between HC and MA cohorts, then that might indicate substantial structural changes and alterations of the brain. The lack of topographical differences among study cohorts supports the notion that mental disorders are more manifested in disruption of brain network dynamics rather than structural changes. Taken together, the similarity in EEG-ms topographies between HC and MA cohorts may suggest that the effect of the depression and anxiety is far more pronounced at the level of dynamic functional connectivity of the brain, rather than at the level of structural abnormalities of the brain.

Next, we appraised EEG-ms average duration and occurrence frequency in our cohorts, as these properties have been used frequently in the literature to differentiate groups (Yuan et al., 2018). Our spatially independent EEG-ms analysis revealed a trend towards significant difference for an average duration of microstate C (*p* < 0.092 corrected for multiple comparisons using the Bonferroni-Holm method). The results did not reveal any other significant difference for average duration or occurrence frequency properties among groups. Furthermore, we analyzed the transition probabilities among different microstate classes for both groups. The analysis showed significant differences in transition probabilities in 4 out of 12 connections in the transition matrices across groups. Specifically, the Tr (D → B) and Tr (B → D) showed statistically significant differences between groups (*p* < 0.05, Bonferroni-Holm corrected), where MA subjects have a lower transition probabilities between Tr (B → D) and Tr (D → B) as compared to HC subjects. That is, MA subjects tend to have a lower switching frequency between microstate B and D as compared to HC subjects. Also, the results revealed a significant difference in transition probabilities for Tr (A → D) and Tr (B → C) in one direction (*p* < 0.05, Bonferroni-Holm corrected), where MA subjects tend to have a higher transition from A → D and B → C. Such disturbances in transition between microstates have been reported for subjects with other mental disorders like schizophrenia (Lehmann et al., 2005) and frontotemporal dementia (Nishida et al., 2013) using traditional EEG-ms analysis.

To understand the results, we refer to outstanding works that investigated the association between EEG-ms and RSNs by using simultaneous EEG-fMRI, where they suggested a strong association between EEG-ms and RSNs (Britz et al., 2010; Musso et al., 2010; Yuan et al., 2012, 2018). However, it is difficult to compare those works with each other and with our studies, since they applied different approaches to extract the EEG-ms. Thus, we rely on Britz et al. (2010) for interpreting our results since the authors utilized the conventional approach in extracting EEG-ms as used in this work. Please refer to Supplementary Table S2 for detailed information about the association between each microstate and RSNs.

Based on Britz et al. (2010), microstate B was shown to be associated with the visual network (VN), while microstate D was related to dorsal attention network (DAN). The associated networks with EEG microstates were similar to the RSNs found in other works (Damoiseaux et al., 2006; Mantini et al., 2007). DAN is often considered as an activity-modulating network in the VN, especially the frontoparietal areas (Vossel et al., 2014). The low transition probabilities between microstate B and D in the MA group indicates less frequent transitions between VN and DAN. The previous study showed a modulatory role of DAN with VN (Vossel et al., 2014), and the impaired modulatory role of DAN might cause less frequent transitions among MA subjects. Several studies reported an alteration in functional connectivity between the two networks for subjects with post-traumatic stress disorder (PTSD; Yin et al., 2012; Gong et al., 2014; Kennis et al., 2016; Zhang et al., 2016), stress (Soares et al., 2013), anxiety (He et al., 2016) and social anxiety disorder (Liao et al., 2010). Furthermore, DAN appears to exhibit an alteration in the functional connectivity associated with depression as reported in the meta-analyses (Wang et al., 2012; Sundermann et al., 2014; Kaiser et al., 2015) and in a recent study (Sambataro et al., 2017). Thus, lower transition probability between B and D may indicate aberrant functionalities between DAN and VN.

In addition, MA subjects exhibit a higher TR (B → C) in one direction. We also noted that MA subjects spend on average more time in microstate C than HC ones (Figure 2). Microstate C has been shown to be correlated with the brain regions responsible for the self-referential mental activity (e.g., parts of DMN). An increase in the self-referential processes in DMN has been shown to be closely related to depression (Lemogne et al., 2009; Sheline et al., 2009). Along with an increase in the average duration of microstate C and the higher transition from microstate B to C, the result may be explained by an increase in the self-referential activity for MA subject with engaging VN in recalling visual memories.

Similarly, MA subjects have a higher TR (A → D) in one direction. Brain regions associated with microstate A have been shown to be involved in the auditory-phonological system, especially the bilateral superior temporal cortex. Such alteration in this RSN has been reported in the meta-analyses for subjects with depression (Wang et al., 2012; Sundermann et al., 2014; Kaiser et al., 2015).

Additionally, we studied the association between the four significant transition probabilities and other clinical assessments (Supplementary Table S3). The results showed a relative correlation between transition probabilities after combining both groups, but not when considering groups independently. However, the transition probabilities showed different patterns based on the group. To further investigate the interaction between groups and symptoms, we designed a generalized linear model (GLM) to study the interaction between groups and symptoms after controlling for age and gender (Supplementary Table S4). The results suggest a significant interaction between groups and symptoms in connections B → D and D → B for PHQ, STAI (State), STAI (Trait) and PROMIS (Anxiety Total Score). These results may imply that HC and MA groups behave differently based on the symptoms, but the relation between symptoms and transition probabilities within groups is more complicated to be explained by one connection.

While the transition matrices unravel the overall behavior of microstates, AIF charactersatics of EEG-ms may encompass an insight into the dynamics of EEG-ms. To do so, we adopted the approach introduced by von Wegner et al. (2017).

Our results were in line with their results that there is a short-term memory effect in EEG-ms sequence as shown in Table 1. For both groups, EEG-ms do not exhibit any Markovian property of order 0, 1, or 2 [i.e., the appearance of next microstate (in time) does not rely merely on the current state, previous state or two previous microstates]. If microstate sequence exhibits any low Markovian order, then one can conclude that microstates appearance relies on the past (depending on the order) only. This demonstrates that microstates embody the underlying neural activities and closely associated with brain activity (Van de Ville et al., 2010). Furthermore, non-Markovian properties show that the sequence has memory.

In addition, our analysis suggested a difference in the information flow manifested in changes of the symmetry and stationary of transition matrices (taken at different periods), besides AIF contents between HC and MA groups. Specifically, the MA group tends to have a higher ratio of subjects with symmetrical (Table 1) and stationary transition matrices (Figure 5) as compared to HC subjects. This may be interpreted as less flexibility and dynamicity of brain connectivity for MA subjects, where similar patterns of brain activations may be evoked frequently (ruminative or self-referential thoughts).

Likewise, the MA group has a relatively higher overall AIF content as compared to HC one (Figure 6) driven by microstate B (Figure 7). Hence, this might be explained as an increase in the overall temporal dependency in MA subjects and more regular appearance for microstate B (associated with VN).

Given these points, MA subjects exhibit a systematic difference in the way of activating their brain regions reflected by changes in transition probabilities, duration of microstate C and temporal dependencies of microstates.

## Limitation

The work has provided several aspects of analyzing MA as compared to HC. We have shown a significant transition probability difference between groups. However, the underlying neurophysiological mechanism of transition probabilities of microstates is still not clear. The provided interpretations of EEG-ms dynamics properties and their associations with brain networks relied on previous studies that found a correlation between EEG-ms time series and different brain regions to interpret the results. In addition, the study cohort is very heterogeneous, thus understanding specific network abnormalities as reflected by EEG-ms within the MA cohort should warrant future studies with an even larger number of subjects to better characterize individual differences and subtypes of the mood and anxiety disorder cohort. Finally, the AIF approach for analyzing the EEG-ms temporal dynamics revealed a group difference among MA and HC cohorts, but the results need further exploration to provide better mechanistic interpretation.

## Conclusion

This work delved into the spatially independent EEG-ms in a large cohort of mood and anxiety disorders and HC individuals. We replicated previously-reported studies and found four EEG-ms classes (A through D), with no differences among mood and anxiety disorders and healthy individuals, suggesting a lack of significant structural cortical abnormalities among the groups, which would otherwise affect the EEG-ms topographies. We investigated several EEG-ms characteristics between groups in terms of average duration, the frequency of occurrence and the transition matrices. In addition, we extracted various AIF properties between groups to evaluate the temporal dependences of microstates between subjects. The results revealed an alteration in EEG-ms transitions probabilities among microstates; in B → D, D → B, A → D and B → C transitions. In addition, testing the temporal dependencies unveiled an alteration in information flow between groups in different properties. Such properties can be used as biomarkers for mood and anxiety disorders and bases for future interventions.

## Data Availability

The datasets generated for this study are available on request to the corresponding author.

## Author Contributions

All authors contributed significantly to the work regarding conception and design; acquisition, analysis; drafting the work and revised critically; approval of the version to be published, and carrying the responsibility for achieving the accuracy or integrity of any part of the work. The presented analysis utilized data from large psychiatric sample obtained with Tulsa 1000 a naturalistic study protocol for multilevel assessment and outcome prediction.

## Conflict of Interest Statement

The authors declare that the research was conducted in the absence of any commercial or financial relationships that could be construed as a potential conflict of interest.
