# Supplementary material for: EEG Microstates Temporal Dynamics Differentiate Individuals with Mood and Anxiety Disorders From Healthy Subjects
Source: Front Hum Neurosci. 2019 Feb 26;13:56. doi: 10.3389/fnhum.2019.00056 (PMC6399140; doi:10.3389/fnhum.2019.00056)
Supplement: Supplementary file 1 [file Table_1.pdf]

Supplementary materials to:

EEG Microstates Temporal Dynamics Differentiate Individuals with Mood and Anxiety Disorders from Healthy Subjects.

Obada Al Zoubi[1,2], Ahmad Mayeli[1,2], Aki Tsuchiyagaito[1,3,4] , Masaya Misaki[1], Vadim Zotev[1], Tulsa 1000 Investigators, Hazem Refai[2], Martin Paulus[1] and Jerzy Bodurka[1,6#]

[1]Laureate Institute for Brain Research, Tulsa, Oklahoma

[2]Department of Electrical and Computer Engineering, University of Oklahoma, Tulsa, OK

[3]Japan Society for the Promotion Science, Tokyo, Japan

[4]Research Center for Child Development, Chiba University, Japan

[5]Oxley College of Health Sciences, University of Tulsa, Tulsa, Oklahoma.

[6]Stephenson School of Biomedical Engineering, University of Oklahoma, Norman, OK

Tulsa 1000 Investigators: Robin L Aupperle[1,5], Sahib S. Khalsa[1,5], Justin S. Feinstein[1,5] , Jonathan Savitz[1,5], Yoon-Hee Cha[1,5], Rayus Kuplicki[1], Teresa A Victor[1]

Supplemental methods

1. Instruments for clinical symptoms assessments.

1.1 The PHQ-9 (Kroenke et al., 2001) is a 9-question instrument with a 2-week time frame (based on diagnostic criteria of depression from DSM-IV), given to patients in a primary care setting to screen, diagnose and measure the severity of depression in last 2 weeks. Each item on the measure is rated on a 4-point scale ("0" =Not at all) to "3" =nearly every day)). The total score can range from 0 to 27, in which a higher score indicates a greater severity of depression.

1.2 The Rumination Response Scale (RRS) (Treynor et al., 2003) is a 22-question assesses depressive thoughts and responses and focuses on the self, symptoms, and possible causes/consequence of associated mood. Each questions consists of scale ranging from 1 (almost never) to 4 (almost always). The RRS has been shown to be a reliable and valid measure with internal consistency of ($\alpha = 0.93$).

1.3 The State-Trait Anxiety Inventory (STAI) has 20 items for assessing state anxiety and 20 items for trait anxiety (Spielberger, 1983). The State Anxiety Scale (S-Anxiety) screens and measures the current state of anxiety, asking how respondents feel "right now," using questions that measure subjective feelings of apprehension, nervousness, tension, worry, and activation/arousal of the autonomic nervous system. The Trait Anxiety Scale (T-Anxiety) evaluates relatively stable aspects of "anxiety proneness," consisting of general states of calmness, confidence, and security. The higher score indicates greater anxiety.

1.4 The Patient-Reported Outcomes Measurement Information System_Anxiety (PROMIS_Anxiety) questioner includes 29 items with a 7-day time frame and a 5-point scale ("1" = Never and "5" = Always) (Cella et al., 2010;Pilkonis et al., 2011). Comprehensive mixed methods was used for developing the item bank (DeWalt et al., 2007), by focusing on fear, anxious misery, hyperarousal, and some somatic symptoms related to arousal.

1.5 The PROMIS_Depression scale consists of 4 items, and asked participants how often in the last 7 days they had experienced depression, including feeling hopeless, worthless, helpless, or depressed (Cella et al., 2010). These items were scored the same way as PROMIS Anxiety on a 5-point Likert scale ranging from 1 to 5.

2. Generalized Linear Model Analysis

Model Description

Transition probability ~ Group * Symptom + Age + Gender

We ran GLM for each connection (Transition probability or y in the model) and symptom independently using "lme4" package from R (Bates et al., 2014). We reported the estimated coefficient and p-values.

Results are presented in supplementary Table S4.

Table S4: The GLM analysis for the interaction between group and symptoms.

Supplemental tables

1. Demographics

Table S1. Demographic information of the study

	Healthy Control	Mood Anxiety
**Gender**		
Female	28	38
Male	24	23
**Age (years)**		
Range	32 (11)	34 (12)
**Education (level)***		
Levels of Studying	6.73 (1.67)	6.27 (1.62)
**PHQ_9**		
Score	0.73 (1.08)	13.26 (5.05)
**STAI_State**		
Score	26.34 (6.10)	46.11 (11.01)
**STAI_Trait**		
Score	28.65 (7.13)	52.7 (11.51)
**PROMIS_Axiety**		
Total Score	46.55 (7.68)	61.96 (6.64)
**PROMIS_Depress**		
Total Score	44.54 (6.48)	61.06 (7.41)

Note: PHQ-9 = Patient Health Questionnaire-9; STAI = State-Trait Anxiety Inventory; PROMIS = Patient-Reported Outcomes Measurement Information System. Values outside parentheses are means and values in parentheses are standard deviations.

*Levels of Studying are assigned as follows:

- No schooling completed -> 1
- Nursery school -> 1
- Kindergarten -> 1
- Grade 1 through 11 -> 2
- 12th grade (no diploma) -> 3
- Regular high school diploma -> 4
- GED or alternative credential -> 5
- Some college credit, but less than 1 year of college credit -> 6
- 1 or more years of college credit, no degree -> 6
- Associate's degree (for example: AA, AS) -> 7
- Bachelor's degree (for example: BA, BS) -> 8
- Master's degree (for example: MA, MS, MEng, MEd, MSW, MBA) -> 9
- Professional degree beyond a bachelor's degree (for example: MD, DDS, DVM, LLB, JD) -> 10
- Doctorate degree (for example: PhD, EdD) -> 11

2. Studies reporting EEG microstates association with BOLD resting state brain networks

Table S2. EEG-ms and related RSNs reported by previous studies.

Microstate	A	B	C	D
**(Britz et al., 2010)**	RSN 1	RSN 2	RSN 3	RSN 4
**(Mantini et al., 2007)**	RSN 4	RSN 3	RSN 6 (Partially)	RSN 2
**(Damoiseaux et al., 2006)**	RSN ‘G’	RSN ‘E’	-	RSN ‘C’
**Brain Regions Involved**	Best matches regions in auditory-phonological system processing network	Primarily involves Visual Network (VN)	A network that involves medial-ventral prefrontal cortex, the pregenual anterior cingulate, the hypothalamus, and the cerebellum self-referential mental activity	The dorsal attention network mediating(DAN)

Note: RSN = BOLD resting state network.

3. Association between EEG-ms transition probabilities and clinical instruments

Table S3. Correlation results between transition probabilities and subjects' assessment measures. The correlation was estimated after combing both groups.

	**Tr (B → D)**		**Tr (D → B)**		**Tr (B → C)**		**Tr (A→ D)**	
	**r**	**p**	**r**	**p**	**r**	**p**	**r**	**p**
**PHQ_9**	-0.205	0.032	-0.267	0.005	0.251	0.008	0.253	0.008
**RRS**	-0.133	n.s.	-0.363	0.001	0.290	0.002	0.284	0.003
**STAI_State**	-0.245	0.010	-0.333	0.001	0.313	0.001	0.171	n.s.
**STAI_Trait**	-0.182	n.s.	-0.317	0.001	0.260	0.006	0.238	0.012
**PROMIS_Anxiety**	-0.122	n.s.	-0.360	0.001	0.216	0.023	0.261	0.006
**PROMIS_Depress**	-0.149	n.s.	-0.277	0.003	0.233	0.014	0.232	0.015

Note: PHQ-9 = Patient Health Questionnaire-9; RRS = Rumination Response Scale; STAI = State-Trait Anxiety Inventory; PROMIS = Patient-Reported Outcomes Measurement Information System. Tr stands for the transition probabilities between two microstates.

4. The results of GLM analysis modeling group and symptom interactions and accounting for age and gender.

Table S4. The GLM analysis for the interaction between group and symptoms.

Connection	Scale measuring symptom	Group	Symptom	Age	Gender	Interaction (Group and Symptom)
Tr (B → D)	PHQ-9	-0.099	-0.034	-0.002	0.014	0.036
	p-value	***.037***	***.046***	*.113*	*.534*	***.040***
Tr (B → D)	STAI_State	-0.295	-0.008	-0.001	0.015	0.009
	p-value	***.003***	***.004***	*.204*	*.495*	***.005***
Tr (B → D)	STAI_Trait	-0.258	-0.004	-0.001	0.010	0.006
	p-value	***.014***	*.100*	*.149*	*.664*	***.047***
Tr (B → D)	PROMIS_Anxiety	-0.017	0.002	-0.001	0.006	-0.001
	p-value	*.924*	*.297*	*.154*	*.797*	*.698*
Tr (D → B)	PHQ-9	-0.114	-0.018	0.001	0.025	0.019
	p-value	***.022***	*.308*	*.159*	*.311*	*.284*
Tr (D → B)	STAI_State	-0.331	-0.010	0.002	0.030	0.010
	p-value	***.001***	***.001***	*.073*	*.191*	***.003***
Tr (D → B)	STAI_Trait	-0.314	-0.007	0.002	0.025	0.008
	p-value	***.004***	***.006***	*.143*	*.290*	***.010***
Tr (D → B)	PROMIS_Anxiety	-0.388	-0.007	0.002	0.030	0.007
	p-value	***.033***	***.004***	*.073*	*.200*	***.045***

Note: PHQ-9 = Patient Health Questionnaire-9; STAI = State-Trait Anxiety Inventory; PROMIS = Patient-Reported Outcomes Measurement Information System. Tr stands for the transition probabilities between two microstates. Numbers in upper row indicate beta coefficient of GLM model and bold numbers in lower row indicate significant *p*-values.

Reference

Bates, D., Maechler, M., Bolker, B., and Walker, S. (2014). lme4: Linear mixed-effects models using Eigen and S4. *R package version* 1**,** 1-23.

Britz, J., Van De Ville, D., and Michel, C.M. (2010). BOLD correlates of EEG topography reveal rapid resting-state network dynamics. *Neuroimage* 52**,** 1162-1170.

Cella, D., Riley, W., Stone, A., Rothrock, N., Reeve, B., Yount, S., Amtmann, D., Bode, R., Buysse, D., and Choi, S. (2010). The Patient-Reported Outcomes Measurement Information System (PROMIS) developed and tested its first wave of adult self-reported health outcome item banks: 2005–2008. *Journal of clinical epidemiology* 63**,** 1179-1194.

Damoiseaux, J., Rombouts, S., Barkhof, F., Scheltens, P., Stam, C., Smith, S.M., and Beckmann, C. (2006). Consistent resting-state networks across healthy subjects. *Proceedings of the national academy of sciences* 103**,** 13848-13853.

Dewalt, D.A., Rothrock, N., Yount, S., and Stone, A.A. (2007). Evaluation of item candidates: the PROMIS qualitative item review. *Medical care* 45**,** S12.

Kroenke, K., Spitzer, R.L., and Williams, J.B. (2001). The PHQ-9: validity of a brief depression severity measure. *Journal of general internal medicine* 16**,** 606-613.

Mantini, D., Perrucci, M.G., Del Gratta, C., Romani, G.L., and Corbetta, M. (2007). Electrophysiological signatures of resting state networks in the human brain. *Proceedings of the National Academy of Sciences* 104**,** 13170-13175.

Pilkonis, P.A., Choi, S.W., Reise, S.P., Stover, A.M., Riley, W.T., Cella, D., and Group, P.C. (2011). Item banks for measuring emotional distress from the Patient-Reported Outcomes Measurement Information System (PROMIS®): depression, anxiety, and anger. *Assessment* 18**,** 263-283.

Spielberger, C.D. (1983). Manual for the State-Trait Anxiety Inventory STAI (form Y)(" self-evaluation questionnaire").

Treynor, W., Gonzalez, R., and Nolen-Hoeksema, S. (2003). Rumination reconsidered: A psychometric analysis. *Cognitive therapy and research* 27**,** 247-259.
